# Neuroinflammation in the peripheral nerve: Cause, modulator, or bystander in peripheral neuropathies?

**DOI:** 10.1002/glia.22899

**Published:** 2015-08-06

**Authors:** Rudolf Martini, Hugh Willison

**Affiliations:** ^1^ Department of NeurologyDevelopmental Neurobiology, University Hospital WürzburgWürzburgD‐97080Germany; ^2^ Institute of Infection, Immunity and Inflammation College of Medical Veterinary and Life Sciences, Glasgow Biomedical Research Centre, University of GlasgowGlasgowG12 8TA

**Keywords:** innate immune system, adaptive immune system, macrophage, fibroblast, lymphocytes, nodes of Ranvier

## Abstract

The role of innate and adaptive inflammation as a primary driver or modifier of neuropathy in premorbidly normal nerves, and as a critical player in amplifying neuropathies of other known causes (e.g., genetic, metabolic) is incompletely understood and under‐researched, despite unmet clinical need. Also, cellular and humoral components of the adaptive and innate immune system are substantial disease modifying agents in the context of neuropathies and, at least in some neuropathies, there is an identified tight interrelationship between both compartments of the immune system. Additionally, the quadruple relationship between Schwann cell, axon, macrophage, and endoneurial fibroblast, with their diverse membrane bound and soluble signalling systems, forms a distinct focus for investigation in nerve diseases with inflammation secondary to Schwann cell mutations and possibly others. Identification of key immunological effector pathways that amplify neuropathic features and associated clinical symptomatology including pain should lead to realistic and timely possibilities for translatable therapeutic interventions using existing immunomodulators, alongside the development of novel therapeutic targets. GLIA 2016;64:475–486

## Introduction

The peripheral nervous system is organized in highly specialized anatomical and functional compartments in which neuronal cell bodies in spinal cord or the dorsal root and autonomic ganglia project the longest known mammalian axons to specialized sensory receptors and muscle synapses. Schwann cells directly encase axons throughout their length, and within the broader nerve microenvironment many other cell types including endothelial cells, pericytes, fibroblasts, and macrophages provide support, surveillance, and likely still undetected functions for proper structural and functional maintenance of the peripheral nerve. The extraordinary physiological complexity including maintenance and homeostasis of this system in both normal and disease states involves major input from the immune system. In this review, we consider the disease states in which immunological activity acts as a primary driver of pathological processes in autoimmune states or modifies neuropathies of other causes. Although there are some commonalities between the distinct disorders (e. g., primary inflammation‐mediated neuropathies vs. secondary inflammation of metabolically or genetically preperturbed nerves), there are also clear differences with regard of the molecular, cellular, and subcellular pathomechanisms. These pathomechanistic details may be important when designing novel treatment approaches. In contrast, the advent of sophisticated and targeted immunotherapies, as now widely used in multiple sclerosis, could provide a chance for treating inflammatory neuropathy of primary or secondary origin.

## Innate and Adaptive Inflammation as a Primary Driver of Neuropathy in Premorbidly Normal Nerves

### Guillain–Barré Syndromes

One of the clearest examples of a disease state in which abnormal immune activation is the primary driver of a destructive pathological process in nerve is the group of autoimmune neuropathies referred to as the Guillain–Barré syndromes (GBS; van den Berg et al., 2014). These acute disorders, reaching their clinical nadir within 4 weeks of onset, are most usually driven by primary immune responses to preceding infections that are inadvertently autoreactive and target nerve plasma membrane components. In some cases, molecular mimicry between microbial and nerve glycans has been formally demonstrated to be the central component of the immunopathological reaction (Willison, 2014). As the primary immune response decays, or is actively suppressed by restoration of immune tolerance, the acute neuroinflammatory phase of the disorder resolves and regenerative pathways take over. The clinical spectrum varies from a mild disorder, in which ambulation is maintained, to a state of complete paralysis, requiring mechanical ventilation. In both extremes, good recovery may occur as remyelination of denuded axons takes effect, provided the extent of irreversible axonal injury is limited. While the driving immune factors in the acute inflammatory phase are destructive, immune cell clearance of myelin, and axonal debris resulting from direct injury and/or Wallerian degeneration may be important for the tissue repair process to be optimally effective (Cashman and Hoke, 2015; Martini et al., 2013).

**Figure 1:**
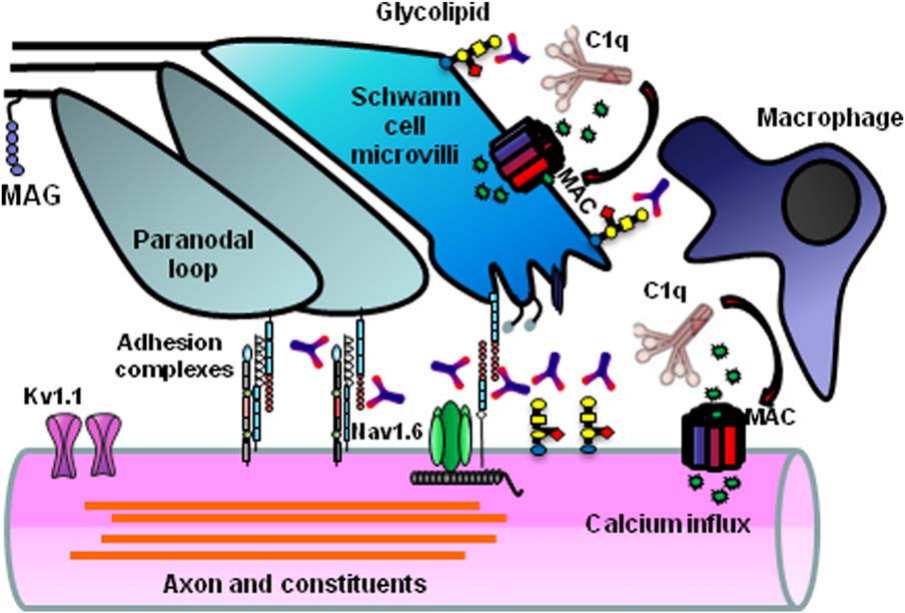
Schwann cell and axolemmal plasma membrane components within the nodal complex, including glycolipids and cell adhesion molecules act as antigens for autoantibodies in acute and chronic autoimmune neuropathy phenotypes. Antibody binding activates the classical complement cascade resulting in deposition of membrane attack complex (MAC). Calcium influx through MAC pores activates calpain and injures vulnerable membranes whose function depends upon both structural integrity (maintained by glial–axonal adhesion complexes) and ion homeostasis (maintained in part by Nav1.6 and Kv1.1 channels). Noncomplement fixing IgG4 subclass antibodies may disrupt local architecture through blocking effects. Soluble complement products and other inflammatory factors recruit macrophages to the injury site.

Historically, GBS was considered to be a single disorder; however, it is now appreciated that within GBS, immune attack may be directed at either myelin/Schwann cell membrane components or axonal components. These subdivisions are termed acute inflammatory demyelinating polyneuropathy (AIDP) and acute motor axonal neuropathy (AMAN). The immunopathology of AIDP and AMAN are quite distinct, the former being characterized by macrophage‐mediated myelin stripping and the latter by primary axonal injury. A new conceptualization of the dichotomization into AIDP and AMAN is the evolving idea of the nodo‐paranodopathy as a more unifying categorization (Uncini and Kuwabara, 2015). The definitive immunopathological study on AMAN was conducted on spinal roots collected at autopsy by Griffin and coworkers (1996). Degenerating motor fibers were observed in the absence of demyelination, with extensive Wallerian degeneration extending beyond the ventral root. Some motor fibers had lengthening of the node of Ranvier accompanied by condensation of the axonal cytoplasm. Infiltrating macrophages extended extensive processes into the periaxonal space abutting the nodal and internodal axolemma and displacing the adaxonal Schwann cell membrane and myelin sheath. Intense IgG and complement C3d and C5b‐9 (membrane attack complex) deposits bound to the nodal and internodal axolemma in the periaxonal space (Fig. 1).

Parallel studies conducted on human AMAN serum samples around the time of these pathological observations revealed autoantibodies to ganglioside components of axonal membranes, notably GM1 and GD1a (Ho et al., 1999; Willison and Yuki, 2002; Yuki et al., 1990). The anti‐ganglioside autoantibodies were found to also react with components of *Campylobacter jejuni* lipo‐oligosaccharide (LOS), this being the major preceding infection in AMAN cases. From these and related studies, it became clear that the immunological cascade in the AMAN form of GBS started with enteric infection by an appropriately glycosylated strain of *C. jejuni*. This induced an anti‐LOS antibody response that, as a consequence of molecular mimicry, also bound to identical epitopes on neuronal gangliosides, resulting in the tissue perturbation described above. From this example, it is thus quite clear that normal nerve can be severely injured by immune attack as a primary event. While the evidence for a similar postinfectious immunological cascade in AIDP is less well understood, it seems likely that ongoing research will uncover closely analogous pathomechanisms.

### Chronic Inflammatory Demyelinating Polyneuropathy and Related Chronic Disorders

In contrast to the notion of GBS as a postinfectious self‐limiting disorder, chronic forms of peripheral nerve inflammation have a less clear relationship to infectious precedents but nevertheless appear due to primary autoimmune attack on otherwise normal nerve. Chronic inflammatory demyelinating polyneuropathy (CIDP) is the prototypic disorder, manifested by motor and sensory deficits (Mathey et al., 2015). Like GBS, it also invariably occurs in premorbidly normal nerves, or evolves subacutely as an extension of unresolved GBS. Support for an autoimmune basis comes from many threads of evidence, including biomarker studies, nerve pathology, and responsiveness to immunotherapy; however, the immunological triggers and subsequent cascade is poorly understood and highly heterogeneous. Two curious antecedents merit mention. First, CIDP may occasionally occur following hematopoietic stem cell transplantation complicated by graft versus host disease (Cocito et al., 2015) and second, following anti‐tumor necrosis factor‐alpha (TNF‐α) antibody therapy as commonly used to treat rheumatoid arthritis (Yagita et al., 2013). While the detailed underlying regulatory mechanisms remain unclear, immune reprogramming and dysfunction of networks maintaining tolerance are likely involved.

Recent biomarkers studies in CIDP have identified autoantibodies to cell adhesion molecules within the nodal complex in a small number of CIDP cases, notably contactin‐1 and neurofascin (Lim et al., 2014; Ng et al., 2012; Querol et al., 2013). The contactin/Caspr/NF155 complex is critical for the formation and maintenance of paranodal junctions. Some evidence suggests these antibodies may be directed against mannose rich N‐glycans within the extracellular domain of contactin, and may interfere within the interaction with NF155, thereby altering paranodal stability (Doppler et al., 2015; Labasque et al., 2014). In this situation, a functional blockade mediated by autoantibodies may be more important than the induction of proinflammatory pathways. Since in at least some of these cases, noncomplement fixing IgG4 antibodies predominate, it will be important to dissect out the downstream pathways leading to conduction failure, as this will dictate the choice of therapies. What is clearly emerging is that CIDP involves highly heterogeneous autoimmune mechanisms, both clinically and immunopathologically (Beppu et al., 2015).

In other chronic inflammatory neuropathies, including multifocal motor neuropathy and paraproteinemic neuropathy, IgM antibodies to nerve glycolipids are frequently found, the targets being the HNK‐1 glycan on myelin‐associated glycoprotein whose epitope is also shared by sulfated glucoronyl paragloboside, and many gangliosides including GM1, GM2, and the disialosyl‐bearing gangliosides GD1b, GD3, GT1b, and GQ1b. As these antibodies are generally of IgM class and potent complement fixing molecules, it is generally believed they act through promoting inflammation at their target membranes; however, they may also exert deleterious crosslinking or blocking effects, for example, trkC‐mediated signalling in dorsal root ganglion neurons (Kusunoki et al., 1999; Takada et al., 2008).

The permanently elevated IgM anti‐glycolipid antibodies seen in these chronic disorders, in contrast to short‐lived IgG antibodies of the same specificities in the acute disorders, may be secreted by long‐lived plasma cells encoding anti‐carbohydrate antibodies within the innate antibody repertoire. Whether such B cells have encountered bacterial antigen‐like *C. jejuni* LOS, or expanded spontaneously, remains to be proven, but the anti‐GM1 antibodies, at least, are certainly capable of binding LOS (Willison and Goodyear, 2013).

### Spontaneous and Induced Animal Models in Which Inflammation Is the Primary Driver of Neuropathy

Historically, the prototypic model of GBS has been the induced rodent disorder, experimental allergic neuritis (EAN), the peripheral nerve counterpart of experimental allergic encephalomyelitis (Soliven, 2014). Many protocols have evolved over the 50 years of its use in rats, mice, guinea pigs, and rabbits and provided extensive information on immunopathological mechanisms that may be relevant to specific facets of human disease (Gold et al., 2000; Maurer et al., 2002). Active immunization with peripheral nerve components including whole myelin or major myelin proteins P0, P2, and PMP22, or with a wide range of glycolipids (e.g., GalC) and gangliosides (e.g., GM1), adoptive transfer with myelin antigen‐specific T‐cells, or passive immunization with specific antibodies and antisera, alone or in combinations have all yielded highly interesting models with unique immunological and clinical features. Recent studies, for example, show critical regulatory roles for FoxP3+ T‐cells in disease severity of several models of EAN (Meyer zu Horste et al., 2014). How closely these models map onto human disease is of great interest and varies from case to case according to species and strain, and immunization parameters. In some, such as sensory ataxic neuropathy induced by anti‐GD1b antibody in the rabbit, the link is very clear as the presence of anti‐GD1b antibodies associates with ataxic neuropathy in man (Yuki and Uncini, 2014). In others such as those induced by adoptive transfer of myelin‐specific T‐cells, the link is less clear since expanded T‐cell repertoires with myelin protein specificity have not been widely identified in man, although recent studies suggest they are present and modified by therapy (Klehmet et al., 2015). What is evident is that a very wide range of nerve‐directed immunological stimuli can induce both acute and chronic neuropathy which, when viewed as models, serve the clinical investigative community well.

Two curious and tragic human immunization events also inform the rodent modelling narrative. First, during the era in which “ganglioside therapy” (i.e., injection of large amount of purified brain gangliosides) for a range of miscellaneous disorders was widespread, the incidence of GBS associated with anti‐ganglioside antibodies was increased among ganglioside therapy recipients relative to the general population (Illa et al., 1995; Odaka et al., 2000). The prevailing conclusion was that inadvertent immunization with intramuscularly injected gangliosides induced anti‐ganglioside antibody‐mediated neuropathy. Another human immunization event comprised an outbreak of autoimmune neuropathy affecting abattoir workers exposed by inhalation to pig brain aerosols liberated during animal carcass processing (Tracy and Dyck, 2011). In essence, these workers received nasal immunization with brain proteins and consequently developed autoimmunity with amplified T‐ and B‐cell responses to multiple brain and nerve antigens. One of the dominant antigens was the voltage‐gated potassium channel complex, a finding which resonates with the sensory hyperexcitability symptoms of pain and paresthesias experienced by the workers. These observations made in humans with unwanted exposure to putative autoantigens were subsequently taken full circle by immunizing mice with the same pig brain aerosol and observing a similar immunological and clinical profile (Meeusen et al., 2012). Both these examples serve to illustrate that immunization of humans with nerve components can clearly induce anti‐nerve autoimmune responses, and thereby, injure the target organ, in the same way that occurs in experimental animals, as modeled in EAN.

In addition to induced models, a spontaneous autoimmune polyneuropathy resembling CIDP occurs in nonobese diabetic mice that lack the T‐cell costimulatory molecule B7‐2 (Soliven, 2011). In these mice, the myelin protein P0‐specific CD4^+^Th1 cell repertoire is expanded and infiltrates the nerve where a demyelinating pathology ensues. The model is widely used to explore basic mechanisms of tolerance and underlying regulatory T‐cell repertoires (Meyer zu Horste et al., 2014). Although pathologically resembling CIDP, the mechanistic link to man is not established.

### Antigen‐Targeted Inflammation in Specialized Nerve Compartments

As indicated above, one feature of human autoimmune neuropathies is the exquisite targeting of very specific nerve compartments, fiber types or anatomical regions. Such selective targeting is expressed clinically as a syndrome manifested by purely motor, sensory, or autonomic features, or motor features restricted to one anatomical site, while sparing others. This is a certain testament to the highly complex organization of peripheral nerves in terms of the distribution of molecular components (capable of acting as antigens) that contribute to its diverse structure and physiology, and the access that cellular and soluble immune factors have to those components. For example, molecules within the nodal complex, when acting as antigens, will attract autoinflammatory injury to that site, as occurs with antibodies directed at the nodal adhesion molecules such as neurofascin 186, contactin‐associated protein 1, and contactin 1 (Querol et al., 2013). The clinical phenotype in CIDP cases associated with anti‐contactin 1 antibodies in one recent study includes sensory ataxia as the predominant presenting clinical feature, suggesting that large dorsal root ganglion neurons and their projecting myelinated axons may be preferentially disrupted, despite the widespread presence of contactin‐1 in all myelinated fibers (Miura et al., 2015). In mice, deficient in contactin‐1 in which paranodal dysfunction is present, ataxia is also a prominent feature, although this may be due to aberrant cerebellar circuitry, rather than loss of peripheral nerve afferent input (Boyle et al., 2001). Conversely, anti‐GM1 antibodies cause predominantly weakness without sensory loss, through targeting axolemmal GM1 in motor nerve fibers, despite the ubiquitous presence of GM1 in all nerve fibers (Willison, 2014). Another striking example of selective regional injury is seen in the Miller Fisher syndrome, in which the anti‐GQ1b antibodies do not injure motor nerves in the limbs but preferentially affect motor nerves innervating extraocular muscles, presumably because GQ1b is enriched in this latter site (Chiba et al., 1997; Liu et al., 2009). The immunopathological basis underlying much of this diversity is unknown but reveals important principles, both clinically and fundamentally.

### Inflammation and Neuropathic Pain

A frequent, nerve inflammation‐related feature is neuropathic pain due to aberrant activation of nociceptive fibers. Pain is a major factor reducing quality of life and is frequently under‐recognized and poorly managed (Liu et al., 2015). While a traditional view would be that the major neuropathic pain syndromes result from metabolic (e.g., diabetes, alcohol toxicity), toxic (e.g., chemotherapy), or genetic (e.g., NaV channel mutations, Fabry's disease, amyloidosis) causes, the contribution of inflammatory mediators to these pathologies is unclear, but may be substantial. Furthermore neuropathic pain is also a major feature of more obviously inflammatory neuropathies of diverse causes. Studies in animal models have identified the release of proinflammatory cytokines (TNF‐α, IL‐1α, IL‐1β, and IL‐6) from cells of both the innate and adaptive immune system as well as from nonimmune cells (Andratsch et al., 2009; Uceyler and Sommer, 2008). Particularly the role of Schwann cells to express TNF‐α during nerve injury is worthwhile to emphasize as mediator for pain induction and maintenance, potentially by directly activating nociceptors (Sorkin et al., 1997). Anti‐inflammatory molecules, such as erythropoietin and the competitive TNF‐α inhibitor etanercept are associated with recovery from neuropathic pain states, underscoring the link between pain and inflammation (Campana et al., 2006; Uceyler and Sommer, 2008).

The situation is complicated by experimental findings that neuropathic stimuli are not only inducing painful inflammation in the nerve but also lead to pain‐related activation of microglia in the spinal cord (Gao and Ji, 2010; Orita et al., 2013; Walters, 2014). It is presently difficult to estimate the individual contributions of inflammation in the peripheral nervous system (PNS) versus in the central nervous system in outcome. According to our view, the overarching problem with pain and inflammation is that it is difficult to predict whether in a particular nerve disorder inflammation is painful or not, as exemplified by painful and painless CIDPs, painful and painless diabetic neuropathies (DNs), and so forth. These issues are underresearched and cannot be extended in this article.

## Models for Inherited Peripheral Neuropathies as Paradigm for Disease Amplification by Inflammation

While the aforementioned neuropathies are primarily driven by the immune system perturbing initially intact peripheral nerves, inherited neuropathies are primarily caused by gene mutations predominantly related to the nervous system. However, there is ample evidence that inflammation substantially contributes to the disease outcome by amplification of the genetically mediated disorders.

Regarding the genetical bases of the inherited neuropathies, the three most common forms have been identified as resulting from mutations in the Schwann cell‐related myelin genes for PMP22, Cx32 (GJB1), and P0 (MPZ) (Suter and Snipes, 1995). Then, particularly the human genome project substantially fueled the detection of further culprit genes (Timmerman et al., 2014), comprising genes affecting cell biological processes of glial cells and neurons at various levels (Berger et al., 2006). Assorting the various disease types follows the Charcot–Marie–Tooth (CMT) classification, dividing the disorders into demyelinating (CMT1) and axonal subtypes (CMT2), but also considering clinical parameters such as disease onset. Most forms show clinical commonalities such as progressive distal muscle weakness, muscular atrophy, and sensory dysfunction, mostly being downstream features of axonal degeneration. Of particular relevance is that up to date no therapies are available (Jerath and Shy, 2014).

Numerous genetically modified rodent CMT models of have been generated (Fledrich et al., 2012). In models mimicking CMT1A (most common), CMT1X (second most common), and CMT1B (third most common), achieved by mild overexpression of peripheral myelin protein 22 (PMP22tg mice; Huxley et al., 1998), deficiency for connexin 32 (Cx32def mice; Anzini et al., 1997) and heterozygous deficiency for myelin protein zero (P0het mice; Giese et al., 1992; Martini et al., 1995), respectively, the mutations in these mice cause some characteristic, dedifferentiation‐related features typical for each model (Klein et al., 2014). However, there are also remarkable similarities among the distinct mutants, with inflammation being a pathomechanistic commonality determining the ultimate outcome of the disease (Groh et al., 2015b).

The aforementioned models share two important cytokine pathways that are strongly related to nerve inflammation and pathogenesis in inherited neuropathies: CCL2 and CSF‐1. Expressed by mutant Schwann cells, CCL2 has been identified as a component to attract and activate pathogenic macrophages in peripheral nerves of CMT1 mutants by cross‐breeding experiments between CCL2‐deficient mice and the animal models (Fischer et al., 2008a; Groh et al., 2010; Kohl et al., 2010a). However, the interpretation of the resulting phenotypes was complicated in that only CMT1‐models being heterozygously deficient for CCL2 showed reduced macrophage numbers and an alleviated phenotype, whereas CMT1‐models completely devoid of CCL2 failed to display macrophage reduction and disease alleviation due to compensatory mechanisms implicating CSF‐1 (Fischer et al., 2008a; Groh et al., 2010; Kohl et al., 2010a). Interestingly, the Schwann cell‐intrinsic mechanism leading from myelin mutation to CCL2 upregulation has been partially deciphered. An important player within this pathway is the MEK‐ERK signalling cascade, as pharmacological blockade of the cascade inhibits CCL2 expression (Fischer et al., 2008b; Groh et al., 2010; Kohl et al., 2010a). Interestingly, this cascade is also activated after nerve injury (Harrisingh et al., 2004), reflecting similarities between inherited neuropathies with Wallerian degeneration (Martini et al., 2013). When the MEK‐ERK cascade is activated via a tamoxifen‐inducible Raf‐kinase transgene in otherwise healthy myelinating Schwann cells, CCL2 expression is induced with inflammation‐related downstream processes, like macrophage activation and demyelination (Napoli et al., 2012). Thus, the MEK‐ERK cascade clearly plays a pivotal role in the diseased peripheral nervous system by linking Schwann cell pathology with detrimental inflammation. Recent studies in an established rat model for CMT1A have hypothesized that the basic culprit in the corresponding disease is the disrupted maturation of the PMP22‐overexpressing Schwann cells due to an imbalance between the PI3K‐Akt and MEK‐ERK pathways (Fledrich et al., 2014). This view was supported by treating the mutants at an early stage of disease with soluble neuregulin‐1 tipping the balance into the direction of PI3K‐Akt, and thus, fostering maturation and improved disease outcome (Fledrich et al., 2014). Whether neuregulin‐1 additionally indirectly attenuated putatively detrimental macrophage activation by blocking the MEK‐ERK cascade would be interesting to investigate in the future (Martini, 2014).

**Figure 2:**
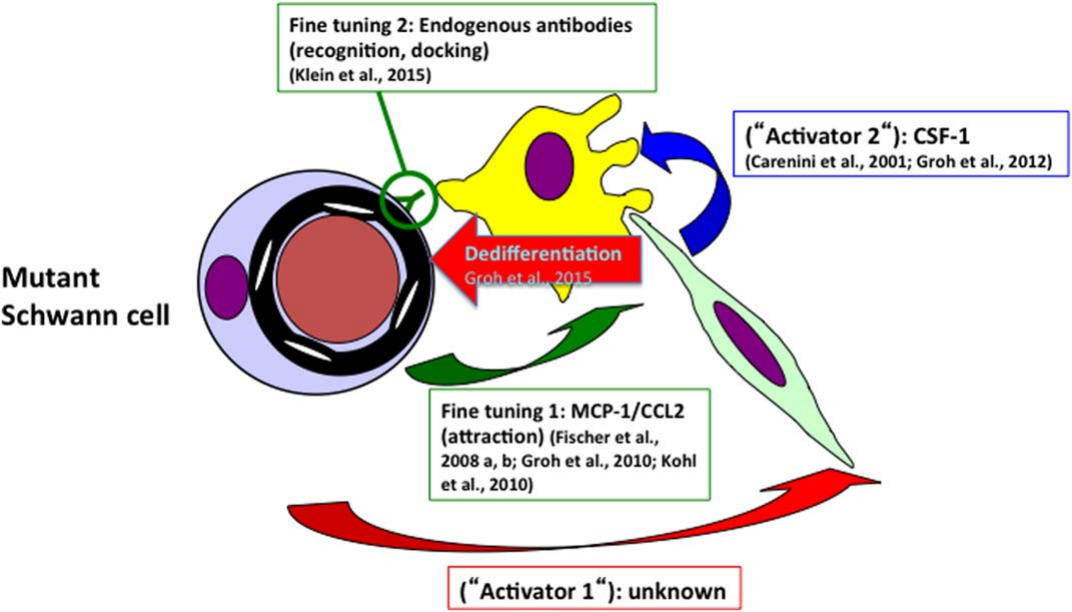
Synoptic view of the interplay of cells and secreted molecules during secondary inflammation in CMT1 models. A not yet identified primary activator (Activator 1) secreted from the mutant Schwann cells induces the endoneurial fibroblasts to express CSF‐1 (Activator 2). CSF‐1 will then activate the macrophages leading to myelin phagocytosis and Schwann cell dedifferentiation, implicating not yet identified signalling molecule(s). Two other, possibly “fine‐tuning” mechanisms have been identified in the form of CCL2 secretion to guide the macrophages to their target (Schwann cells) and the endogenous antibodies binding to mutant Schwann cells and enabling the macrophages to recognize their target, likely by Fc‐receptors. Purple cell: mutant Schwann cell; brownish profile: myelinated axon; yellow cell: macrophage; green cell: endoneurial fibroblast.

While PMP22 overexpression may directly lead to impaired Schwann cell maturation/differentiation causing nerve pathology, a recent study by Groh et al. (2015a) in Cx32def mice proved that a CMT1‐related myelin mutation alone is not sufficient for Schwann cell immaturity/dedifferentiation and nerve pathology. In mice heterozygously deficient for Cx32 and showing a coexistence of Cx32wt and Cx32def Schwann cells in one and the same nerve, pathogenic macrophage clusters were only associated with Cx32def Schwann cells, reflecting a target‐directed behavior of nerve macrophages. It is plausible to assume that under these conditions the pathogenic macrophage clusters may be guided to their targets by CCL2, expressed by the mutant Schwann cells (Groh et al., 2010, 2015a). Most interestingly, when Cx32het, Cx32def, and P0het mice additionally lacked CSF‐1, nerve pathology is robustly and constantly ameliorated (Carenini et al., 2001; Groh et al., 2015a, 2012). Moreover, Schwann cells retained their differentiated, myelinated phenotype although they displayed an activated MEK‐ERK cascade and increased levels of the downstream chemokine CCL2 (Groh et al., 2015a). The scenario is even more complicated by the observation that the potent macrophage activator CSF‐1 is not expressed by Schwann cells, but by endoneurial fibroblasts (Groh et al., 2012; Martini et al., 2013). This leads to the still unresolved question how fibroblasts “sense” that the Schwann cells are mutant (Fig. 2).

In addition to the implication of innate immune cells, there is also evidence for an involvement of the adaptive immune system in models for inherited peripheral neuropathies. In both P0het and Cx32def mice crossbreeding of these myelin mutants with mice deficient in *Rag1* (*recombinase activating gene 1*), lacking mature T‐ and B‐lymphocytes, resulted in a marked amelioration of the demyelinating and axonopathic phenotype and a reduction of macrophage numbers (Kobsar et al., 2003; Schmid et al., 2000). Surprisingly, however, in PMP22tg mice the absence of lymphocytes does not influence nerve pathology (Kohl et al., 2010b), while—as discussed above—macrophages are crucial players in the development of the pathology (Kohl et al., 2010a).

Which lymphocytes are responsible for the disease‐amplifying effect in P0het and Cx32def mice is not yet known. In principle, B‐lymphocytes might be interesting candidates as antibodies produced by plasma cells and decorating nerve fibers could function as potential recognition signals for phagocytizing macrophages as previously shown for Wallerian degeneration (Vargas et al., 2010). Indeed, our recent findings demonstrate that in peripheral nerves of P0het mice, endogenous antibodies of IgG and IgM subtypes decorate endoneurial tubes of peripheral nerves (Klein et al., 2015a). Crossbreeding with JHD−/− mice lacking B‐cells and antibodies, lead to an amelioration of disease similarly as described for P0het/RAG‐1‐deficient mice. Interestingly, lack of evidence for complement activation along the antibody‐labeled fibres and increase of nerve macrophages in antibody‐reconstituted mice suggest an interaction of nerve‐bound antibodies with macrophages, most probably via Fc‐receptors (Klein et al., 2015a). Thus, endogenous antibodies are excellent candidate molecules to link the innate with the adaptive immune system in at least one model of CMT1 neuropathies. However, this view does not exclude the possibility that T‐lymphocytes are also involved which needs further investigations.

In summary, a picture emerged identifying a coordinated interplay between components of both the innate and adaptive immune system as amplifiers of the primarily genetically caused neuropathies (Fig. 2). Within this scenario, a not yet identified primary activator (Activator 1) secreted from the mutant Schwann cells and inducing the endoneurial fibroblasts to express CSF‐1 (Activator 2) has to be postulated. CSF‐1 will then activate the macrophages leading to myelin phagocytosis and Schwann cell dedifferentiation. As described above, 2 other, possibly “fine‐tuning” mechanisms have been identified in the form of CCL2 secretion to guide the macrophages to their target and the endogenous antibodies binding to mutant Schwann cells and enabling the macrophages to recognize their target, likely by Fc‐receptors and possibly others. Interestingly, Fc‐receptors have been identified to be upregulated by macrophages downstream of CSF‐1 activation (Chitu and Stanley, 2006). There is hope that the deciphering of this interaction and further investigations on inflammatory pathways in CMT‐models will provide opportunities for therapeutic options of the so far untreatable disorders.

## Potential Roles of Inflammation in Other Primarily Immune‐Unrelated Neuropathies

### Diabetic Neuropathy

DNs are frequent and serious complications of diabetes mellitus types 1 and 2, preferentially affecting the longest axons of the sensory, motor, and autonomous nervous system (Cashman and Hoke, 2015; Hoke, 2012; Vincent et al., 2011). Remarkably, pathological leading features are axonal degeneration, demyelination, and remyelination (Hoke, 2012; Vincent et al., 2011). In the following, we will briefly focus on insights into the inflammatory pathways of the length‐dependent nerve disorders, a topic that has very recently been reviewed comprehensively by others (Cashman and Hoke, 2015).

The predominant symptoms with a significant, life quality‐reducing impact are sensory perturbations such as pain and paresthesias or numbness, the latter of which can lead to serious complications like ulcerations and amputations. Devastating consequences of the impaired autonomic nervous system are—among others—reduced blood flow in blood vessels which likely amplify the neuropathic features (Cashman and Hoke, 2015; Vincent et al., 2011).

It is most likely that the full‐blown image of DN comprises multiple and complicated pathomechanisms, implicating the polyol pathway, abnormal lipid metabolism, protein glycosylation, radical‐mediated damage, inflammation, and the deficiency of neurotrophic factors (Cashman and Hoke, 2015; Vincent et al., 2011). The majority of these mechanisms have partially been characterized in rodent models like rats and mice, fed with the pancreatoxic compound streptozotocin, a model best representing diabetes type 1 (Hoke, 2012). These models were particularly helpful in deciphering some of the pathomechanisms, but studies in both nerve and skin biopsies were also instrumental (Bekircan‐Kurt et al., 2014; Sommer, 2008).

One of the aforementioned aspects is the reduced glycemic control, which may cause metabolic injury in neurons, partially by means of mitochondrial damage via reactive oxygen species. The link to inflammation is the generation of advanced glycation end products (AGE), that is, organic compounds like proteins, lipids, and nucleic acids that are nonenzymatically modified by reactive carbohydrate groups. These can bind to a cognate cell surface receptor (RAGE; Cashman and Hoke, 2015; Sandireddy et al., 2014; Vincent et al., 2011), a pattern recognition receptor, likely playing a central role in the inflammation‐related pathogenic arm of DN. This receptor is expressed in many different cells and tissues that are potentially involved in immune reactions during DN, including vessels, dermal endothelial cells, monocytes, macrophages, lymphocytes, peripheral epidermal axons, sural axons, Schwann cells, dorsal root ganglia neurons, and others (Bekircan‐Kurt et al., 2014; Lin et al., 2009; Vincent et al., 2011). Most importantly, the inflammation‐related transcription factor NF**‐**kB is activated downstream of RAGE binding, leading to the expression of a plethora of proinflammatory cytokines activating inflammatory cascades with additional detrimental effects, including oxidative stress (Sandireddy et al., 2014; Yan et al., 1994; Yeh et al., 2001). The situation is complicated as the RAGE‐ NF**‐k** B axis is also activated by proinflammatory molecules and potentially by “accidental” ligands (Lin et al., 2009), causing a potential vicious cycle of disease amplification (Vincent et al., 2011). Potential downstream effectors of RAGE activation, such as TNF‐α, have recently been shown to be a disease promoting condition, as TNF‐α receptor blockade using a recombinant human TNF‐α receptor‐antibody fusion protein obviously alleviates streptozotocin‐induced DN in rats (Shi et al., 2013).

Although many experimental data exist, it is presently difficult to get a clear picture of the impact of inflammation in DN. It is plausible to assume that the many different cell types expressing RAGE, the various different ligands, including proinflammatory cytokines (Lin et al., 2009), cause a pathogenic scenario of potentially tremendous complexity of DN‐related inflammation. This may even vary during disease duration and progression. A scenario is emerging in which DN is promoted by the direct inflammatory damage of neural cells or by impaired blood vessels or both. The overall complexity of the inflammatory pathway may also explain that some findings appear controversial and may depend on variations in exact experimental design. For example, RAGE inactivation has been reported to alleviate experimental DN and promote PNS regeneration in some studies (Vincent et al., 2011), but does not support slow axonal transport and regeneration in others (Juranek et al., 2013). Moreover, even the expression pattern of RAGE in nerve and skin biopsies may vary, likely depending on the position of biopsy and/or stage of disease (Bekircan‐Kurt et al., 2014). In summary, it is beyond doubt that more work is urgently needed to systematically unwrap the potentially important role of inflammation in DN, as targeting inflammation may be an option to mitigate DN. Applying novel sophisticated experimental models (e.g., cell‐specific and/or inducible RAGE mutants), thoroughly defining experimental disease stages and using standardized and translatable readout measures in the experimental models are some of the most important improvements for further studies (Hoke, 2012).

Importantly, there are additionally other types of neuropathic disorders that can be typically seen in diabetic patients and are associated with inflammation. For example, nerve length‐independent mononeuropathies and diabetic brachial or lumbosacral plexoneuropathies (with nondiabetic derivatives) are described (Dyck and Giannini, 1996; Dyck and Windebank, 2002), but the underlying inflammatory processes could so far not be molecularly characterized as in the length‐dependent DN, most likely due to lack of animal models.

### Familial Amyloidotic Polyneuropathy

In contrast to DN, familial amyloidotic polyneuropathy (FAP) is a rare neurological disorder. It is associated with the deposition of amyloid fibrils in the endoneurium of the peripheral nervous system and, when untreated, its prognosis is fatal due to serious effects in vital organs (Sousa et al., 2001b; Ueda and Ando, 2014). The disease is mostly associated with point mutations in the transthyretin (TTR) gene, with a Val30Met substitution being the predominant cause (Ueda and Ando, 2014). A link to inflammation has been demonstrated by Yan et al. (2000) and Sousa et al. (2001b), showing an interaction of amyloid fibrils with RAGE causing downstream effects including NF‐kB signalling and inflammation, a surprising commonality with DN. TNF‐α, IL‐1β, CSF‐1 (M‐CSF) have been identified as proinflammatory downstream‐components in FAP biopsies (Sousa et al., 2001b). Unexpectedly, and in contrast to CMT neuropathy and other models, the CSF‐1 was reported to be found in myelinated axons (Sousa et al., 2001a). A similarity to CMT models is the observation that both in patients and transgenic model mice the MEK‐ERK cascade is activated within Schwann cells as possible response to fibrillar deposition (Monteiro et al., 2006). It is not known whether this leads to a Schwann cell‐borne CCL2 expression and macrophage activation as has been shown in CMT—and other transgenic models of neuropathy (Napoli et al., 2012). In a recent study, the group of Saraiva and colleagues report about the impact of IL‐1 during pathogenesis of FAP and show that the disease can be ameliorated in a mouse model by the IL‐1 antagonist Anakinra (Goncalves et al., 2015, 2014). These combined studies suggest that inflammation may play a modifying role in the pathogenesis of FAP.

## Targeting Inflammation as an Opportunity to Make Diseases “Treatable”

Although nerve inflammation is generally known as causing disability and possibly pain in various peripheral nerve disorders, the detailed negative impact of innate and adaptive inflammation on peripheral nerve structure, function, and regeneration is under‐researched and, therefore, poorly understood. There is particular need for novel approaches targeting particular compartments of the immune system. Concerning the primary inflammatory disorders, like CIDP, a recently initiated clinical trial using modern immune modulators, like fingolimod (FTY720), might be promising (Melzer and Meuth, 2014). Current treatment for these conditions is limited to nonspecific immunotherapies such as corticosteroids, broadly acting immunosuppressants like azathiaprine and methotrexate, and intravenous immunoglobulin and plasma exchange, both of which have pleiotropic immune‐modulating effects. The advent of highly targeted immunotherapies, as now widely used in multiple sclerosis, should provide further impetus for clinical trials in inflammatory neuropathy (Nobile‐Orazio and Gallia, 2013).

As for the models for inherited neuropathies, the inflammatory disease pathway has been systematically investigated, some specific options emerge which might be realistic to follow. Beforehand, it has to be approved whether the situations seen in the rodents faithfully reflect the situations in man.

Indeed, there are some observations supporting the view that in human patients similar processes occur as in the mouse models, especially in children with CMT1. Analogous to the mouse models, myelin‐laden foamy macrophages within the endoneurial space can occasionally be detected by electron microscopy (Crawford and Griffin, 1991). In addition, MHC class II positive macrophages can often be found integrated into onion bulb formations (Stoll et al., 1998). Even more striking similarities between diseased nerves in animal models and human biopsies have recently been reported: by the combination of electron microscopy with immunohistochemical approaches not only the presence of myelin‐laden macrophages was detected in both species, but, as a pathogenetically important detail, also contacts of macrophages with fibroblasts expressing CSF‐1 have been found as commonality between biopsies of CMT1 patients and nerves from the corresponding mouse models (Groh et al., 2012). These findings suggest that similar low‐grade inflammatory reactions might occur in the peripheral nerves of CMT1 patients, especially early in pathogenesis. Taken this as a base, it would now be interesting to investigate such issues also in acquired neuropathies (NPs). A caveat might, however, be that (1) animal models for acquired neuropathies may not always be faithful recapitulators of human disease and (2) interrogating human peripheral nerve through tests such as sural nerve biopsies are often not available, or yield insufficient temporal and spatial information to be of high value. However, these could be partially replaced by the less invasive skin biopsies (Nolano et al., 2015) to investigate a potential involvement of macrophages in a respective NP.

A possible approach to inhibit or attenuate the mechanisms related to inflammation have been identified in the genetic mouse models using MEK‐ERK cascade blockers (Fischer et al., 2008b; Groh et al., 2010; Kohl et al., 2010a). Although the effects in the genetic models was substantial, this or related strategies are rather of academic value in CMT models rather than a realistic option in the nonfatal inherited neuropathies, as the MEK‐ERK pathway is pleiotropically involved in a variety of cell biological processes and thus side effects have to be expected by long term inhibition of this pathway. Therefore, it is understandable that this strategy is only considerable for severe malignancies (Rusconi et al., 2012).

Another direction for an immune‐modulatory treatment approach might emerge by interfering with the CSF‐1‐CSF‐1R axis, based on the robust and persistent amelioration of neuropathy by CSF‐1 deficiency in different CMT1 mouse models (Carenini et al., 2001; Groh et al., 2012). A corresponding pharmacological analog might be silencing the CSF‐1‐CSF‐1R axis by using appropriate inhibitors (Elmore et al., 2014). Recently, a corresponding approach indeed led to substantially improved histopathological, physiological, and clinical features in two mouse models of inherited peripheral neuropathies (Klein et al., in press).

Regarding DN, the RAGE‐NF**‐k**B axis leading to TNF‐α, IL‐1β, and CCL2 expression, would theoretically be a possible target for amelioration of inflammation, neuropathic features, and possibly pain. Surprisingly, treatment algorithms for diabetic pain do obviously not consider anti‐inflammatory drugs (Vincent et al., 2011), although TNF‐α can function as mediator of neuropathic pain (Uceyler and Sommer, 2008). As RAGE is also expressed on endothelial cells, use of anti‐inflammatory drugs could theoretically also improve the vascular complications as exemplified in treating diabetic rats with erythropoietin (Vincent et al., 2011). Possibly future experiments will show whether the many immune modulators recently developed for treating multiple sclerosis might be helpful for ameliorating one of the most common forms of neuropathy, DN.

As an inflammation‐related commonality between DN and FAP, the RAGE‐NF**‐k**B axis would, theoretically, also be a target for treatment. However, immunotherapies appear presently not to play a predominant role in trying to treat FAP in the clinics. Rather, the highly invasive liver transplantation is a therapeutic option for patients below the age of 60 and inflammation unrelated drugs, like stabilizers of TTR tetramers (tafamidis and diflunisal) and potentially gene‐therapeutic approaches are novel, noninvasive alternatives for liver transplantation (Adams et al., 2014; Scott, 2014; Sekijima, 2014; Ueda and Ando, 2014).

## Synopsis

In this article, we provide an overview of what is known about the pathogenic impact of inflammation in peripheral neuropathies. Along these lines, we discriminated neuropathies primarily caused by the immune system (GBS, CIDP) versus others in which secondary inflammation emerges as a result of genetic abnormalities (CMTs) or impaired metabolism (DN, FAP). Comparing these two major categories, it appeared that there are inflammation‐related commonalities, but also clear differences between them. For instance, in AMAN and CMTs, at first glance, macrophages appear to be similar promoters of nerve damage when superficially viewed. Upon detailed inspection, in AMAN macrophages slip into nodes of Ranvier and precisely or even exclusively cause axon damage, followed by features comparable of Wallerian degeneration (Griffin et al., 1996). In CMT models, macrophages predominantly associate with the abaxonal aspect of the mutant Schwann cells, lead to juxtaparanodal alterations (Groh et al., 2012) and, in addition to myelin disruption, they promote a CSF‐1‐dependent dedifferentiation pathway of Schwann cells which might be, secondarily, detrimental to axons (Groh et al., 2015a). As this scenario appears to be more related to CSF‐1‐dependent tumor‐associated macrophages that drive proliferation and dedifferentiation of mutant (tumor) cells (Mantovani and Sica, 2010) than to macrophage‐related processes going on in GBS/AMAN, it is a tempting to speculate that pathogenic mechanism within the nerve can strongly vary among the different nerve disorders but can show clear commonalities with disorders not at all related to the nervous system (e.g., cancer). Another issue concerns the role of antibodies. Antibodies specific to nodal, internodal, and presynaptic glycolipids but also against proteins belonging to the nodal complex may either act as epitope‐blocking agents and/or fix complement that lead to cellular damage. Based on a novel study, in the CMTs, endogenous antibodies also play a mild, pathogenic role possibly activating macrophages via Fc‐receptors, as it is similarly observed in Wallerian degeneration (Klein et al., 2015a; Vargas et al., 2010). However, there is lack of evidence for an involvement of complement (Klein et al., 2015a).

Taking into consideration that it is likely that upon closer analysis of neuroinflammatory processes both commonalities but also clear differences will emerge, it is pivotal to thoroughly decipher the pathogenic impact of immune cells very precisely. This could lead to the fortunate situation that pathogenic commonalities to disorders will emerge for which treatment approaches are already available.
